# Oxyresveratrol Supplementation to C57bl/6 Mice Fed with a High-Fat Diet Ameliorates Obesity-Associated Symptoms

**DOI:** 10.3390/nu9020147

**Published:** 2017-02-16

**Authors:** Hui Yuan Tan, Iris Mei Ying Tse, Edmund Tsze Shing Li, Mingfu Wang

**Affiliations:** School of Biological Sciences, The University of Hong Kong, Hong Kong, China; totanhy@gmail.com (H.Y.T.); mytsea@hku.hk (I.M.Y.T.); etsli@hku.hk (E.T.S.L.)

**Keywords:** oxyresveratrol supplementation, amelioration, high-fat diet, obesity, glucose homeostasis, lipid homeostasis

## Abstract

Oxyresveratrol has been proven effective in inhibiting adipogenesis in a 3T3-L1 cell model. We investigated the preventive effect of oxyresveratrol supplementation on obesity development in high-fat diet-fed mice. Male C57bl/6 mice were randomly subjected to control (5% fat by weight, LF), high-fat (30% fat by weight, HF), and high-fat supplemented with 0.25% and 0.5% oxyresveratrol (OXY1 and OXY2, respectively) diet groups for eight weeks. Oxyresveratrol supplementation effectively alleviated obesity-associated symptoms such as insulin resistance, hyperglycemia, and hepatic steatosis in high-fat diet-fed mice. Compared to the high-fat diet group, oxyresveratrol supplementation suppressed expression of glucose-6-phosphatase, sterol regulatory element-binding proteins 1, fatty acid synthase and CCAAT/Enhancer-binding proteins α, and elevated AMP-activated protein kinase (α2-catalytic subunit) level in liver, upregulated insulin-dependent glucose transporter type 4 level in adipose tissue, and increased expression of insulin receptor substrate 1, insulin-dependent glucose transporter type 4, AMP-activated protein kinase α, peroxisome proliferator-activated receptor γ coactivator-1α, and sirtuin 1 in muscle to regulate lipid and glucose homeostasis in these tissues. This study demonstrated that oxyresveratrol supplementation effectively ameliorated obesity-associated symptoms in high-fat diet-fed mice, presumably attributed to mediating critical regulators involved in lipid and glucose homeostasis in liver, visceral fat, and muscle.

## 1. Introduction

**Figure 1:**
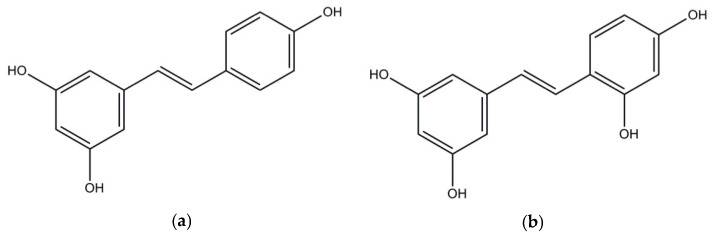
Structures of resveratrol (**a**) and oxyresveratrol (**b**).

Over the years, the prevalence of obesity has been increasing worldwide with the obese population more than doubling since 1980. Around the world, more than 1.9 billion adults were overweight or obese in 2014. Additionally, in 2014, 41 million children under the age of 5 were reported to suffer from overweight or obesity [1]. More and more adults as well as children suffer from abnormal or excessive body fat with a variety of comorbidities, such as hypertension, dyslipidemia, type 2 diabetes, cardiovascular disease, stroke, sleep apnea, knee osteoarthritis, and certain cancers [2,3]. Obesity has been considered a global epidemic disease [1,4]. Soaring demands for effective anti-obesity strategies are driving industry and academia to conduct research in this field. To prevent or heal obesity with the supplementation of phenolic compounds have generated intense interest in recent years. Among the studied phenolics, resveratrol (Figure 1a) has been extensively studied with both in vitro and in vivo studies demonstrating that resveratrol has great potential in the management of obesity. Supplementation of resveratrol is capable of relieving the harmful effects induced by a high-calorie diet such as reducing the rodents body weight gain, adipose tissue depots, plasma triglycerides, and increasing their survival and motor function [5].

Being similar in structure with resveratrol, oxyresveratrol (OXY) (Figure 1b) possesses an additional hydroxyl group on its aromatic ring [6,7]. OXY is a natural polyphenol first isolated from the heartwood of *Artocarpus lakoocha* Roxb and also rich in mulberry (*Morus alba* L.) twigs and woods [8]. Studies on OXY have revealed that it possesses similar biological activities as resveratrol and the potential beneficial effects include anti-inflammatory, anti-oxidative, anti-viral, and neuroprotective activities [9,10,11,12,13,14]. It has also been considered a potent free radical scavenger, a tyrosinase inhibitor, and an anti-browning agent that could be used in food industry for cloudy apple juices and fresh-cut apples [7,15,16].

Our previous study demonstrated that OXY, at non-cytotoxic doses, possesses anti-adipogenic ability in 3T3-L1 cells by inhibition of differentiation through inducing cell cycle arrest [17]. However, little is known about OXY’s impact on obesity in vivo. The present study thus aimed at investigating the preventive effects of OXY supplementation on the development of obesity in mice fed with a high-fat diet. C57bl/6 male mice were randomly assigned to control (5% fat by weight, LF), high-fat (30%, HF), and high-fat supplemented with 0.25% and 0.5% OXY (OXY1 and OXY2, respectively) diet groups for eight weeks. Growth parameters, organ and adipose tissue weights, serum biochemical parameters, and the expressions of relevant mRNA/protein in liver, adipose tissues, and muscles were examined to identify the putative anti-obesity effect of OXY and gain insight on the underlying mechanism.

## 2. Materials and Methods 

### 2.1. Oxyresveratrol and Experimental Diets

OXY (≥98% pure, CAS registry No. 29700-22-9) was purchased from Great Forest Biomedical Ltd., Hangzhou, China. The purity was confirmed by High Performance Liquid Chromatography analysis.

The formulation for the experimental diets was modified based on the AIN-93G recommendation [18]. The control diet was the low-fat diet (LF, 5% fat *w*/*w*) that comprised corn oil (50 g/kg) as lipids. The high-fat diet (HF, 30% fat *w*/*w*) comprised corn oil (150 g/kg) and Crisco shortening (150 g/kg) as lipids. The two treatment groups were the high-fat diets supplemented with 0.25% OXY (OXY1) and 0.5% OXY (OXY2). OXY was added to the high-fat diet at the expense of cornstarch. OXY1 and OXY2 diets contained 2.5 g and 5 g OXY/kg, respectively. Diet compositions with energy density were stated in Table 1. All prepared diets were stored at −40 °C and fresh diets were provided every other day.

### 2.2. Experimental Design

39 male C57bl/6 mice, four weeks of age, were obtained from the animal unit of Faculty of Medicine, The University of Hong Kong, Hong Kong. The animals were housed individually under controlled temperature and 12 h light-dark cycle and had free access to water. The mice were randomly assigned to one of the four diet groups (*n* = 8–11) for eight weeks: low-fat diet (LF, 5% fat *w*/*w*); high-fat diet (HF, 30% fat *w*/*w*); high-fat diets supplemented with 0.25% OXY (OXY1) and 0.5% OXY (OXY2). Food intake and body weight were monitored every other day. At the end of eight weeks, blood was drawn from the abdominal vena cava from each mouse and then the mouse was killed by cervical dislocation. Tissues including liver, visceral fat, and gastrocnemius muscle were collected and stored at −80 °C for assays of biochemical parameters, and gene/protein expression. All the procedures were performed with the approval of the ethics committee on the Use of Live Animals in Teaching and Research at The University of Hong Kong (No. 3263-14).

### 2.3. Measurement of Biochemical Parameters

Serum was prepared by leaving the blood to clot undisturbed at room temperature for 30 min and then removing the clot by centrifuging at 2000 g for 10 min at 4 °C. Serum glucose was determined by Glucose Assay Kit (Abcam, Cambridge, UK). Serum high-density lipoprotein (HDL) cholesterol was assayed by Cholesterol, HDL Test, Precipitating Reagent (Stanbio Laboratory, Boerne, TX, USA). Cholesterol was determined with Cholesterol LiquiColor (Stanbio Laboratory, Boerne, TX, USA). Serum insulin was measured by Mercodia Mouse Insulin ELISA kit (Mercodia, Uppsala, Sweden). The HOMA-IR (homeostasis model assessment of insulin resistance) index was calculated as (fasting serum glucose (mmol/L) × fasting serum insulin (mIU/L)/22.5) to assess insulin resistance [19,20]. Serum and liver triglyceride (TG) were assayed using Triglycerides LiquiColor (Stanbio Laboratory, Boerne, TX, USA). Non-esterified fatty acid (NEFA) was measured by LabAssay NEFA kit (Wako Pure Chemical Industries, Osaka, Japan).

### 2.4. Total RNA Isolation and Real-Time Reverse Transcriptase Polymerase Chain Reaction (PT-PCR) Analysis

Total RNA from liver, visceral fat, and gastrocnemius muscle were extracted with Trizol reagent (Invitrogen, Waltham, MA, USA) following the manufacturer’s instructions. cDNA was synthesized from 0.5 μg of total RNA using iScript Select cDNA Synthesis Kit (Bio-Rad Laboratories, Hercules, CA, USA). cDNA and TaqMan probes were subjected to quantitative Real-Time PCR amplification by TaqMan Universal Master Mix II (Applied Biosystems, Carlsbad, CA, USA) on the StepOnePlus Real-Time PCR System (Applied Biosystems, Carlsbad, CA, USA). The thermal profile settings were 50 °C for 2 min and 95 °C for 10 min and then 40 cycles at 95 °C for 15 s and 60 °C for 1 min. Relative expression levels of the mRNA of the target genes were normalized to GAPDH mRNA levels.

### 2.5. Protein Extraction and Western Blotting Analysis

Mice liver tissue fragments were homogenized in an ice cold lysis buffer (25 mM 4-(2-hydroxyethyl)-1-piperazineethanesulfonic acid (pH 7.5), 150 mM sodium chloride, 1 mM ethylenediaminetetraacetic acid disodium salt, 1 mM dithiothreitol, 1% Triton X-100) with added 4% protease inhibitor cocktail, 1% phosphatase inhibitor cocktail B, and 2% phosphatase inhibitor cocktail C for protein collection. Protein concentration in samples was determined using the Bradford Reagent (Bio-Rad Protein Assay Dye Reagent Concentrate). 30 µg of protein of each sample was applied for electrophoresis on 12% sodium dodecyl sulfate polyacrylamide gel electrophoresis gels and transferred to polyvinylidene diflouride membrane. Membranes were blocked with 5% nonfat dried milk powder in phosphate buffered saline (PBS) solution with tween-20 or tris buffered saline (TBS) solution with tween-20 (0.1% *v*/*v* tween-20 in PBS or TBS) overnight at 4 °C. The membranes were then incubated for 2 h with specific antibodies at room temperature. Equal sample loading was verified by anti-β-actin. Membranes were developed using SuperSignal West Pico Chemiluminescent Substrate (Pierce Biotechnology, Rockford, IL, USA). The bands were quantified densitometrically using the software ImageJ 1.47v (Wayne Rasband, Bethesda, MD, USA).

### 2.6. Statistical Analysis

The data were presented as means ± S.E.M. (the standard error of the mean). One-way ANOVA with Duncan corrections was used to determine significance for multiple comparisons. For all analyses, the accepted level of significance was *p* < 0.05. The calculations were performed using SPSS statistical package version 11.0 for Windows (International Business Machines Corporation, Armonk, NY, USA).

## 3. Results

### 3.1. Effects of Oxyresveratrol on Body Weight, Energy Intake, Energy Efficiency, and Tissue Weights

**Figure 2:**
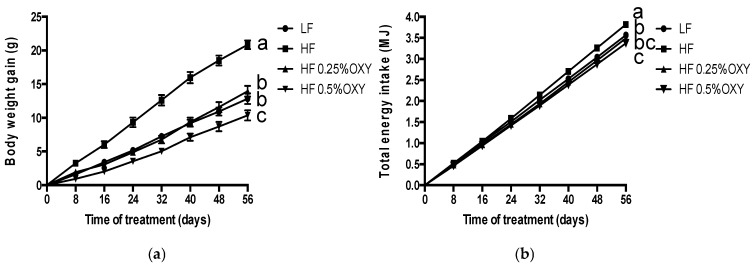
Effects of oxyresveratrol supplementation on body weight gain (**a**) and total energy intake (**b**). C57bl/6 mice were fed low-fat or high-fat diet with or without oxyresveratrol (*n* = 8–11/group) for eight weeks. Values are presented as means ± S.E.M. Means by different letters are significantly different at *p* < 0.05.

After feeding with designated diets for eight weeks, the HF mice had significantly higher body weight gain, energy intake and energy efficiency than that of the LF mice (Figure 2, Table 2, *p* < 0.05). While, OXY supplemented high-fat feeding mice (OXY1, OXY2) had significantly reduced body weight gain, energy intake and energy efficiency as compared to those of the HF mice (Figure 2, Table 2, *p* < 0.05). Comparing the two OXY supplemented groups, both body weight gain and energy efficiency were significantly lower in the OXY2 group, while energy intakes were not significantly different (Figure 2, Table 2, *p* < 0.05). Furthermore, as indicated in Table 2, there was no significant difference in gastrocnemius muscle weights among the four groups, while the weights of liver and visceral fat in OXY1 and OXY2 mice were significantly reduced as compared to those of the HF mice (Table 2, *p* < 0.05).

### 3.2. Effects of Oxyresveratrol on Serum Concentration of Glucose and Insulin

As shown in Table 3, the serum concentrations of glucose and insulin in HF mice were significantly higher than those of the LF mice; the OXY supplementation significantly suppressed the levels of these two parameters in serum of high-fat fed mice, and the serum insulin level was decreased to the level of the LF mice (Table 3, *p* < 0.05). Compared to that of other three groups, the HOMA-IR index for the high fat group was significantly increased. Meanwhile, no difference in the HOMA-IR index was observed among the OXY supplemented mice and the LF mice (Table 3, *p* < 0.05).

### 3.3. Effects of Oxyresveratrol on Lipid Profiles in Serum and Liver

Compared to the HF group, 0.5% OXY supplementation to a high-fat diet for eight weeks significantly lowered serum levels of TG, cholesterol, and NEFA and also liver levels of TG and cholesterol (Table 3, *p* < 0.05).

### 3.4. Effects of Oxyresveratrol on mRNA Expression of Liver, Visceral Fat, and Gastrocnemius Muscle

**Figure 3:**
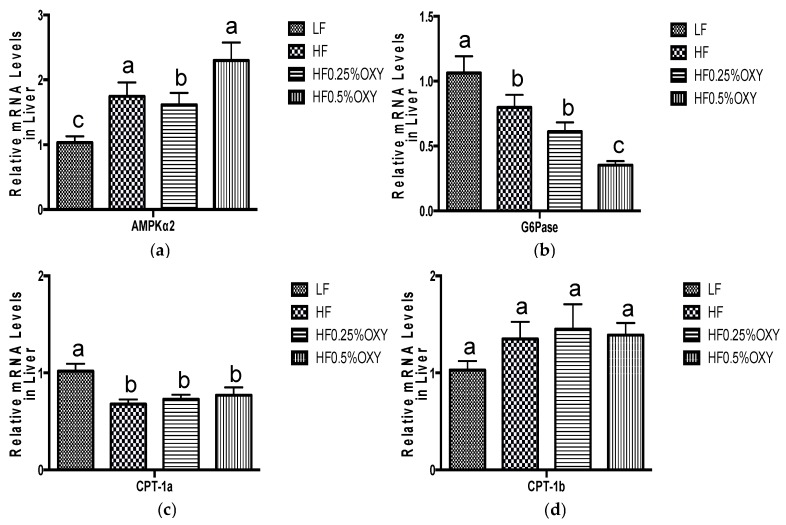
Relative mRNA expression levels of AMPKα2 (**a**), G6Pase (**b**), CPT-1a (**c**), and CPT-1b (**d**) in liver of C57bl/6 mice that were fed low-fat or high-fat diet with or without oxyresveratrol (*n* = 8–11/group) for eight weeks. Values are presented as means ± S.E.M. Within each treatment, bars topped by different letters are significantly different at *p* < 0.05.

**Figure 4:**
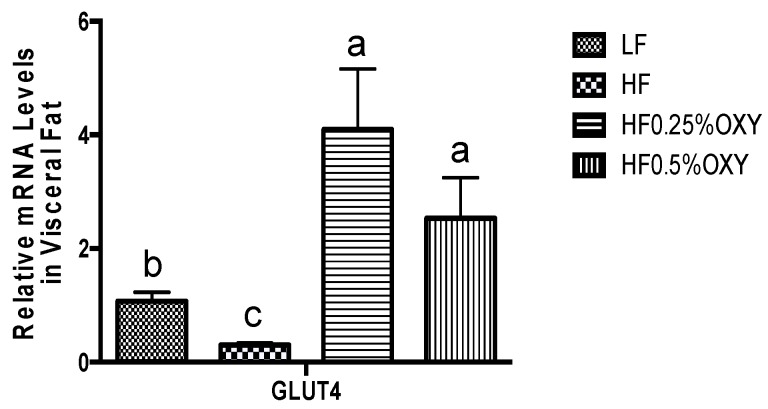
Relative mRNA expression level of GLUT4 in visceral fat of C57bl/6 mice that were fed low-fat or high-fat diet with or without oxyresveratrol (*n* = 8–11/group) for eight weeks. Values are presented as means ± S.E.M. Within each treatment, bars topped by different letters are significantly different at *p* < 0.05.

**Figure 5:**
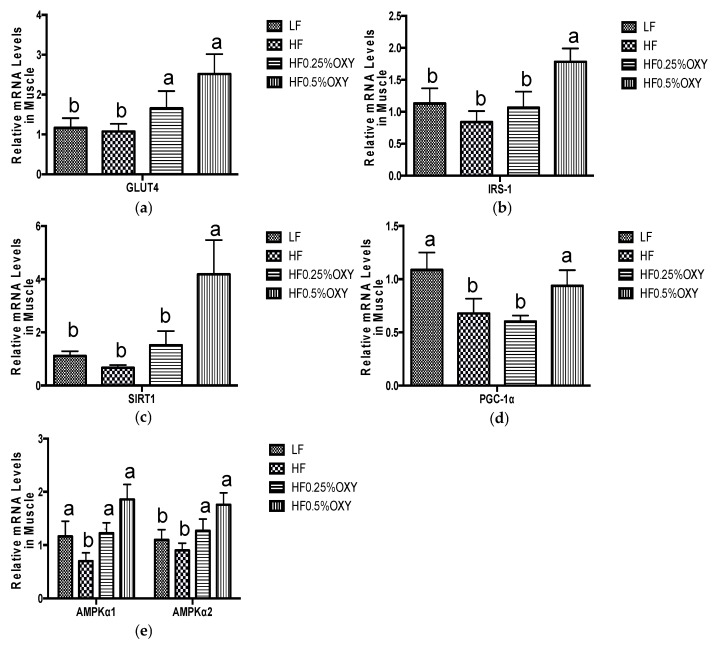
Relative mRNA expression levels of GLUT4 (**a**), IRS1 (**b**), SIRT1 (**c**), PGC-1α (**d**), AMPKα1 (**e**), and AMPKα2 (**e**) in gastrocnemius muscle of C57bl/6 mice that were fed low-fat or high-fat diet with or without oxyresveratrol (*n* = 8–11/group) for eight weeks. Values are presented as means ± S.E.M. Within each treatment, bars topped by different letters are significantly different at *p* < 0.05.

In OXY supplemented HF groups (especially in OXY2 group), hepatic mRNA expression of AMP-activated protein kinase α2 (AMPKα2) was upregulated, whilst glucose-6-phosphatase (G6pase) level was downregulated compared to the HF group (Figure 3a,b). Hepatic mRNA expressions of carnitine palmitoyltransferase IA (CPT-1a), carnitine palmitoyltransferase IB (CPT-1b) in oxyresveratrol OXY groups were similar to those of the HF group (Figure 3c,d). Mice fed with a high-fat diet for eight weeks had decreased mRNA expressions of glucose transporter type 4 (GLUT4) in visceral fat, whereas GLUT4 level was upregulated in OXY supplemented HF groups (Figure 4). In gastrocnemius muscle, mRNA expressions of GLUT4, insulin receptor substrate 1 (IRS-1), SIRT1, peroxisome proliferator-activated receptor gamma coactivator 1-alpha (PGC-1α), AMPKα1 and AMPKα2 were all upregulated in OXY supplemented groups (especially in OXY2) compared to the HF group (Figure 5).

### 3.5. Effects of Oxyresveratrol on Hepatic Protein Expression

**Figure 6:**
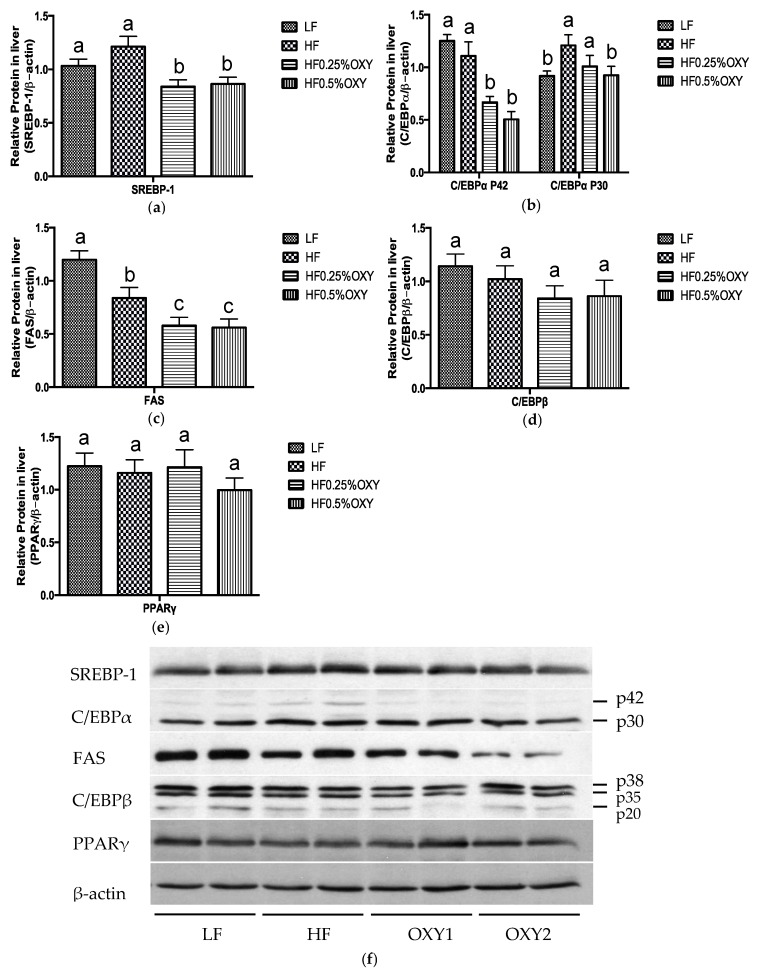
Protein expressions of SREBP-1 (**a**,**f**), C/EBPα (**b**,**f**), FAS (**c**,**f**), C/EBPβ (**d**,**f**), and PPARγ (**e**,**f**) in liver of C57bl/6 mice that were fed low-fat or high-fat diet with or without oxyresveratrol (*n* = 8–11/group) for eight weeks. Protein expressions were quantified densitometrically using the software ImageJ 1.47v (Wayne Rasband, Bethesda, MD, USA), and were arbitrary units after correction for loading differences by measuring the amount of β-actin. Values are presented as means ± S.E.M. Within each treatment, bars topped by different letters are significantly different at *p* < 0.05.

In liver, protein levels of sterol regulatory element-binding proteins 1 (SREBP-1), CCAAT/Enhancer-binding proteins α (C/EBPα), and fatty acid synthase (FAS) were significantly downregulated in OXY supplemented high-fat diets groups (Figure 6a–c,f). While no significant difference on CCAAT/Enhancer-binding proteins β (C/EBPβ) and peroxisome proliferator-activated receptor γ (PPARγ) has been observed among the four mice groups (Figure 6d–f).

## 4. Discussion

In recent years, resveratrol has been demonstrated to effectively lower body weight and adiposity in rodents fed a high-calorie diet by inducing favorable changes in specific gene and protein expression [5]. Being similar in structure with resveratrol, OXY has been identified to hold various biological functions that are comparable to those of resveratrol. However, little is known about its anti-obesity property. Our previous in vitro study determined that OXY possesses anti-adipogenic property in 3T3-L1 cells by regulating some key transcriptional factors, as well as inducing cell cycle arrest through modulation of specific cell cycle regulatory molecules [17], which encouraged us to study the potential effect of OXY in regulating obesity in vivo. In the present study, OXY was evaluated of its impact on weight management, obesity-related biochemical parameters, and the regulation of adiposity related gene and protein expression in high-fat diet-fed mice. The results clearly manifested that, compared to the HF group, OXY supplementation remarkably induced suppression of body weight (up to 26%), and organ weight of liver (up to 28%), and visceral fat (up to 51%), restored serum level of glucose and insulin, and ameliorated the lipid profile of serum and liver in high-fat diet-fed mice.

Diet composition is closely related to energy efficiency. Mice with high-fat diet induced obese would have increased energy efficiency, partly resulting from the high energy content of triglycerides, which are stored in essentially anhydrous form other than the hydrated form for polysaccharides, reducing the energy stores’ efficiency as fuel [21,22,23]. In this study, an increase in energy efficiency has been observed in the HF group, while OXY1 diet (2.5 g OXY/kg HF diet) decreased energy efficiency in mice and normalized it to that of the low-fat diet group. Moreover, comparing to the OXY1 group, mice fed with OXY2 diet (5 g OXY/kg HF diet) had significantly lower body weight gain, energy efficiency, and visceral fat weight without changing the energy intake, which suggested that there might be a dose dependent effect for OXY to regulate obesity.

Insulin, a peptide hormone secreted by the β cells in the pancreas, functions to suppress serum level of glucose by catalyzing glucose transfer into adipose tissue and muscle, and reducing hepatic glucose production [24]. Obesity development could lead to insulin resistance, characterized by the raised level of circulating insulin, as well as suppressed insulin sensitivity [25,26]. In this study, serum insulin level and insulin resistance index, HOMA-IR, in the HF group mice was significantly elevated compared to those of the LF group, while OXY supplementation alleviated them in high-fat diet-fed mice and reduced their levels to those of the LF mice.

Under the condition of insulin resistance, cells in insulin dependent tissues, principally adipose tissues and muscles, are resistant to insulin and fail to respond to it effectively, inducing a high level of serum glucose [27]. In this study, serum glucose level was significantly elevated in the HF group compared to that of the LF group, while OXY supplementation significantly reduced its level. For adipocytes and muscle cells, intracellular glucose uptake is insulin-dependent via GLUT4, a major glucose transporter protein. Adipose tissue is supposed to account for around 10% of insulin-regulated whole body glucose uptake [27]. Through GLUT4, excess blood glucose is diffused into adipocytes, stimulating the synthesis of fatty acid and glycerol, while suppressing lipolysis. Similar with what happens to adipocytes, the transportation of intracellular glucose into muscle cells is also via GLUT4 under the regulation of insulin. Muscle is the primary site of insulin-stimulated glucose disposal, and accounts for around 60%–70% of whole body glucose uptake [27,28]. Earlier researches suggested that GLUT4 gene expression is decreased in adipose tissue, while retained in muscle under various insulin resistant states [28]. With the increased GLUT4 expression in adipose tissue or muscle, or both, glucose tolerance and insulin sensitivity could be elevated in normal, obese, or diabetic mice [28]. In this study, the mRNA expression of GLUT4 was downregulated in visceral fat, but preserved in muscles of the HF group compared to that of the LF group, and the results are consistent with those previous studies. Nevertheless, the OXY supplementation significantly upregulated GLUT4 expression in both visceral fat tissue and muscle of high-fat fed mice, which might stimulate insulin sensitivity, increasing blood glucose uptake into adipocytes and muscle cells, reducing serum glucose level in OXY supplemented mice groups.

Apart from GLUT4, IRS-1—a member of the insulin receptor substrate family of adaptor molecules—also plays vital role in intracellular glucose uptake. It is involved in insulin signaling through tyrosine phosphorylation in response to insulin, insulin growth factor-1, and cytokines [29]. The reduced insulin receptor and IRS-1 levels, their depressed tyrosine phosphorylation together with the diminished IRS1-associated phosphatidylinositol-3 kinase activity all devote to the defective insulin-stimulated glucose transport through impairing GLUT4 translocation in muscle in obese subjects [30,31]. In this study, mRNA levels of IRS-1 and GLUT4 were both significantly increased in muscle of OXY treated group (OXY2) compared to the HF group, suggesting that OXY supplementation might increase glucose uptake and disposal in muscle through ameliorating insulin resistance by stimulating IRS-1 and GLUT4 expression.

The decreased mRNA expression of hepatic G6Pase in the OXY supplemented groups might also due to the ameliorated hyperglycemia. G6Pase is a multifunctional enzyme capable of hydrolyzing glucose-6-phosphate, and producing free glucose, which is released from liver to blood [32]. The downregulation of G6Pase in OXY supplemented groups might result in the reduced production of hepatic glucose, thus lowering the release of glucose into the bloodstream. The above results demonstrated that hyperglycemia induced by high-fat diet in mice could be effectively alleviated by OXY supplementation, presumably attributed to OXY’s ameliorative effect in insulin resistance by elevating glucose uptake into peripheral tissues and depressing hepatic glycogenolysis.

During obesity development, the defective hepatic metabolism as well as the excessive adiposity lead to an increase in plasma free fatty acids level [24]. In this study, serum triglyceride, cholesterol, and NEFA levels, as well as hepatic triglyceride and cholesterol levels were significantly lowered in OXY supplemented group (OXY2) compared to the HF group. In liver, the protein expressions of SREBP-1 and FAS were both diminished in OXY supplemented groups than those of the HF group. SREBP-1, a member of the basic helix-loop-helix leucine zipper family, regulates triglyceride synthesis through mediating the transcription and expression of lipogenic proteins, such as FAS, which is a multi-functional enzyme mainly involved in catalyzing the synthesis of long chain fatty acids [33,34]. OXY supplementation might reduce fat content in liver through the SREBP-1 pathway by suppressing SREBP-1 expression and, hence diminishing FAS activity to lower fatty acid and triglyceride synthesis and storage.

Meanwhile, the hepatic expression of C/EBPα protein was suppressed in OXY supplemented groups compared to the HF group. C/EBPs are members of the basic leucine zipper family of transcriptional factors and mainly function by directing the adipose-specific gene expression during adipocyte transition process [35,36]. C/EBPα is primarily expressed in hepatocytes and adipocytes and has been well documented to involve in energy homeostasis [37,38]. The deficiency of hepatic C/EBPα could decrease body lipid level by diminishing the induction of lipogenic genes to reduce hepatic TG and cholesterol concentration [38]. Therefore, apart from the SREBP-1 pathway, OXY may also restrict hepatic lipid synthesis through the downregulation of hepatic C/EBPα.

Also, hepatic AMPKα2 expression was elevated in OXY supplemented groups. AMPK is an energy sensing enzyme that plays a vital role in regulating metabolic homeostasis through mediating mitochondrial biogenesis in response to energy deprivation [39]. AMPKα is a subunit of AMPK with two isoforms—namely, AMPKα1 and AMPKα2 [31]. AMPKα1 is widely expressed, whereas AMPKα2 is the dominant catalytic form of AMPK in the liver, muscle, and hypothalamus and essential for the modulation of metabolic homeostasis and insulin sensitivity [40]. In liver, AMPK is capable of suppressing hepatic glucose, free fatty acid, TG, and cholesterol synthesis by inactivating specific gene and protein expressions in gluconeogenesis and lipogenesis [31]. In this study, the increased hepatic AMPKα2 expression thus may be a result of the reduced hepatic glucose and lipids synthesis. Therefore, OXY may ameliorate hepatic fat accumulation mainly through mediating the expression of adipogenic gene/proteins (SREBP-1, FAS, C/EBPα, and AMPKα2). The alleviated hepatic metabolism together with the reduced adiposity in OXY supplemented groups may work collaboratively to regulate lipid profile in serum.

In the OXY supplemented group (OXY2), muscle mRNA expressions of AMPKα, PGC-1α, and SIRT1 were all upregulated compared to the HF group. PGC-1α, a metabolic co-activator, functions in stimulating mitochondrial biogenesis and respiration through interacting with transcription factors [41]. It controls fiber-type switching and the expression of various genes involved in lipid oxidation and mitochondrial metabolism [42]. AMPK can activate PGC-1α by direct phosphorylation, enhancing its transcriptional activity [39]. Meanwhile, AMPK needs PGC-1α to regulate the expression of several important genes involved in mitochondrial and glucose metabolism [42]. SIRT1, which is well documented for its beneficial effect in life span extension, can also interact with PGC-1α in muscle directly by deacetylation, promoting mitochondrial activity, thus improving exercise performance and thermogenic activity [42,43]. In muscle, AMPK and SIRT1 work cooperatively to mediate energy metabolism, and AMPK could enhance SIRT1 activity through upregulating cellular nicotinamide adenine dinucleotide level, leading to the deacetylation and regulation of the activity of downstream SIRT1 targets [39]. A recent in vivo study indicated that AMPK, PGC-1α, and SIRT1 act as an energy sensing network to promote metabolic fitness, suggesting that OXY supplementation might benefit metabolic homeostasis through stimulating the expression of AMPK, PGC-1α, and SIRT1 in muscle to advance their network [42]. However, further studies are needed to verify their connections and effects on obesity development.

## 5. Conclusions

OXY supplementation remarkably induced suppression on body weight (up to 26%), organ weight of liver (up to 28%), and visceral fat (up to 51%). It restored serum level of glucose and insulin, which was presumably through increasing intracellular glucose uptake in muscle and adipose tissue by upregulating expression of key glucose transportation genes (GLUT4/IRS1) in these tissues, as well as repressing free glucose production in liver by suppressing hepatic G6Pase expression. Meanwhile, lipid profile of serum and liver was significantly ameliorated in OXY supplemented high-fat diet-fed groups, which was presumably through mediating expression of major adipogenic genes/proteins (SREBP-1, FAS, C/EBPα, AMPKα2) in liver. Also, OXY might promote metabolic fitness in high-fat diet-fed mice by elevating the expressions of AMPKα, PGC-1α, and SIRT1 in muscle. To our knowledge, this is the first study providing in vivo evidence for OXY’s anti-obesity property. Further research in other animal models or in humans should be considered to verify OXY’s effect in counteracting obesity.

## Figures and Tables

**Table 1:** Composition of the experimental diets ^1^.

Ingredient	LF ^2^	HF ^3^	OXY1 ^4^	OXY2 ^5^
			g/kg	
Casein ^6^, 87.5%	200	235	235	235
Corn starch ^6^	549.5	255.5	253	250.5
Sucrose ^6^	100	100	100	100
Cellulose (fiber) ^6^	50	50	50	50
Crisco ^7^	0	150	150	150
Corn oil ^8^	50	150	145	145
Oxyresveratrol	0	0	2.5	5
Mineral ^6^, AIN-93G-MX	35	42	42	42
Vitamin ^6^, AIN-93-VM	10	12	12	12
Choline bitartrate	2.5	2.5	2.5	2.5
l-Cystine	3	3	3	3
Tert-butylhydroquinone	0.014	0.014	0.014	0.014
Energy ^9^, kJ/g	15.69	20.69	20.46	20.42

^1^ Based on AIN-93G diet with modification. ^2^ LF: Low-fat diet. ^3^ HF: High-fat diet. ^4^ OXY1: High-fat diet supplemented with 0.25% oxyresveratrol. ^5^ OXY2: High-fat diet supplemented with 0.5% oxyresveratrol. ^6^ Harlan Teklad (Madison, WI, USA). ^7^ Crisco, partially hydrogenated vegetable shortening (Procter & Gamble, Orrville, OH, USA). ^8^ Mazola (CPC, Kuala Lumpur, Malaysia). ^9^ Based on energy densities: 16.74 kJ/g for protein and carbohydrates and 37.66 kJ/g for fat.

**Table 2:** Effects of oxyresveratrol supplementation on body weight, energy intake, energy efficiency, and tissue weights of mice ^1,2^.

Parameters	LF ^3^	HF ^4^	OXY1 ^5^	OXY2 ^6^
**Body weight**				
Starting body weight, g	19.61 ± 0.21	19.71 ± 0.20	19.56 ± 0.25	19.56 ± 0.31
Final body weight, g	32.46 ± 0.84 ^b^	40.54 ± 0.71 ^a^	33.51 ± 0.82 ^b^	29.92 ± 0.93 ^c^
Body weight gain, g	12.85 ± 0.81 ^b^	20.83 ± 0.67 ^a^	13.95 ± 0.79 ^b^	10.35 ± 0.77 ^c^
**Energy intake**				
Fat intake, MJ	0.43 ± 0.01 ^c^	2.09 ± 0.02 ^a^	1.89 ± 0.02 ^b^	1.84 ± 0.05 ^b^
Energy intake, MJ	3.57 ± 0.05 ^b^	3.82 ±0.04 ^a^	3.49 ± 0.04 ^bc^	3.37 ± 0.09 ^c^
Energy efficiency ^7^, g/MJ	3.60 ± 0.00 ^b^	5.45 ± 0.00 ^a^	3.98 ± 0.00 ^b^	3.04 ± 0.00 ^c^
**Tissue weights**				
Liver, g	1.12 ± 0.04 ^b^	1.34 ± 0.07 ^a^	1.08 ± 0.04 ^bc^	0.96 ± 0.04 ^c^
Visceral fat, g	1.75 ± 0.13 ^c^	3.31 ± 0.07 ^a^	2.35 ± 0.15 ^b^	1.60 ± 0.15 ^c^
Gastrocnemius muscle, g	0.31 ± 0.03	0.29 ± 0.02	0.29 ± 0.01	0.30 ± 0.01

^1^ Values are mean ± S.E.M. *n* = 8–11 per group at eight weeks. ^2^ Means in each row with superscripts without a common letter differ, *p* < 0.05. ^3^ LF: Low-fat diet. ^4^ HF: High-fat diet. ^5^ OXY1: High-fat diet supplemented with 0.25% oxyresveratrol. ^6^ OXY2: High-fat diet supplemented with 0.5% oxyresveratrol. ^7^ Energy efficiency: body weight gain (g)/energy intake (MJ). Energy efficiency is calculated as described by Li et al. [21].

**Table 3:** Effects of oxyresveratrol supplementation on serum and liver biological parameters of mice ^1,2^.

Biological Parameters	LF ^3^	HF ^4^	OXY1 ^5^	OXY2 ^6^
**Serum**				
Glucose, mM	4.8 ± 0.4 ^c^	9.6 ± 0.8 ^a^	6.7 ± 0.6 ^b^	7.2 ± 0.3 ^b^
Insulin, μg/L	0.5 ± 0.3 ^b^	1.5 ± 0.3 ^a^	0.4 ± 0.1 ^b^	0.3 ± 0.2 ^b^
HOMA-IR index ^7^	4.4 ± 1.7 ^b^	20.1 ± 4.6 ^a^	4.7 ± 1.2 ^b^	3.2 ± 1.4 ^b^
Triglyceride, mg/dL	132.7 ± 5.8 ^a^	110.4 ± 7.4 ^b^	105.3 ± 5.3 ^b^	84.6 ± 5.9 ^c^
Cholesterol, mg/dL	139.7 ± 4.9 ^ab^	153.4 ± 3.3 ^a^	142.3 ± 4.9 ^ab^	134.0 ± 4.7 ^b^
HDL cholesterol, mg/dL	89.4 ± 2.7	92.7 ± 1.0	91.4 ± 2.0	86.8 ± 3.0
NEFA, mEq/L	1.7 ± 0.1 ^a^	1.5 ± 0.1 ^ab^	1.5 ± 0.1 ^b^	1.1 ± 0.1 ^c^
**Hepatic lipids**				
Triglyceride, mg/g liver	14.1 ± 0.4 ^a^	14.1 ± 0.5 ^a^	12.9 ± 0.3 ^ab^	11.9 ± 0.5 ^b^
Cholesterol, mg/g liver	2.5 ± 0.2 ^a^	2.4 ± 0.1 ^a^	1.8 ± 0.1 ^b^	1.7 ± 0.1 ^b^
NEFA, 10^−3^ mEq/g liver	3.6 ± 0.2 ^b^	4.6 ± 0.3 ^ab^	4.9 ± 0.4 ^ab^	5.6 ± 0.7 ^a^

^1^ Values are mean ± S.E.M. *n* = 8–11 per group at eight weeks. ^2^ Means in each row with superscripts without a common letter differ, *p* < 0.05. ^3^ LF: Low-fat diet. ^4^ HF: High-fat diet. ^5^ OXY1: High-fat diet supplemented with 0.25% oxyresveratrol. ^6^ OXY2: High-fat diet supplemented with 0.5% oxyresveratrol. ^7^ HOMA-IR index: fasting serum glucose (mmol/L) × fasting serum insulin (mIU/L)/22.5.
